# Monocyte Migration Driven by Galectin-3 Occurs through Distinct Mechanisms Involving Selective Interactions with the Extracellular Matrix

**DOI:** 10.1155/2013/259256

**Published:** 2013-02-25

**Authors:** Cláudia Danella Polli, Karina Alves Toledo, Luís Henrique Franco, Vânia Sammartino Mariano, Leandro Licursi de Oliveira, Emerson Soares Bernardes, Maria Cristina Roque-Barreira, Gabriela Pereira-da-Silva

**Affiliations:** ^1^Programa de pós-graduação em Imunologia Básica e Aplicada, FMRP/USP, Ribeirão Preto, SP, Brazil; ^2^Faculdade de Ciências e Letras, Universidade Estadual Paulista (UNESP), Campus de Assis, São Paulo, SP, Brazil; ^3^Departamento de Biologia Celular e Molecular e Bioagentes Patogênicos, FMRP/USP, Ribeirão Preto, SP, Brazil; ^4^Departamento de Biologia Geral, UFV, Viçosa, MG, Brazil; ^5^Centro de Investigação Translacional em Oncologia, Instituto do Câncer do Estado de São Paulo, São Paulo, SP, Brazil; ^6^Departamento de Enfermagem Materno-Infantil e Saúde Pública, EERP/USP, 3900-14040-902 Ribeirão Preto, SP, Brazil

## Abstract

Monocyte migration into tissues, an important event in inflammation, requires an intricate interplay between determinants on cell surfaces and extracellular matrix (ECM). Galectin-3 is able to modulate cell-ECM interactions and is an important mediator of inflammation. In this study, we sought to investigate whether interactions established between galectin-3 and ECM glycoproteins are involved in monocyte migration, given that the mechanisms by which monocytes move across the endothelium and through the extravascular tissue are poorly understood. Using the *in vitro* transwell system, we demonstrated that monocyte migration was potentiated in the presence of galectin-3 plus laminin or fibronectin, but not vitronectin, and was dependent on the carbohydrate recognition domain of the lectin. Only galectin-3-fibronectin combinations potentiated the migration of monocyte-derived macrophages. In binding assays, galectin-3 did not bind to fibronectin, whereas both the full-length and the truncated forms of the lectin, which retains carbohydrate binding ability, were able to bind to laminin. Our results show that monocytes migrate through distinct mechanisms and selective interactions with the extracellular matrix driven by galectin-3. We suggest that the lectin may bridge monocytes to laminin and may also activate these cells, resulting in the positive regulation of other adhesion molecules and cell adhesion to fibronectin.

## 1. Introduction

The migration of peripheral circulating monocytes into tissues is considered an important process in the immune response, including inflammatory reactions. During this process, monocytes migrate from the circulation, across the endothelium and the basement membrane, and into the affected tissue. When monocytes enter tissues, they eventually mature into macrophages to carry out their main functions. As reported concerning other leukocytes, the monocyte migratory response occurs through a multistep adhesion mechanism. Interactions established between leukocytes and extracellular matrix (ECM) components are crucial to the cell migration through the perivascular tissue [1]. 

Lectins may mediate many of the adhesive interactions during leukocyte extravasation and directly influence the migratory response of these cells [2, 3]. Galectin-3, a *β*-galactoside-binding lectin belonging to the galectin family, is involved in many processes during the inflammatory response [4]. This lectin is expressed and secreted by various inflammatory cells [4] and induces migration of monocytes/macrophages [5]. The mechanisms by which galectin-3 induces monocyte/macrophage migration are not fully elucidated, and those involving interactions with the ECM have not been addressed to date.

Galectin-3 is composed of a carboxyl-terminal carbohydrate recognition domain (CRD) and aminoterminal tandem repeats. Upon binding to its glycan ligands at the cell surface, galectin-3 oligomerizes by self-assembly of its N-terminal regulatory domain [4], a characteristic that makes it suited for a cell adhesion function, including cell-to-cell and cell-to-extracellular matrix protein interactions [4]. Indeed, a role in the traversing of neutrophils through the basement membrane at inflammation sites has been suggested for this lectin [4].

Although a great deal of effort has led to the characterization of the initial steps of the leukocyte migration process, considerably less is known concerning the movement of leukocytes across the endothelium and through the extravascular tissue [1]. In the present study, we sought to investigate whether interactions established between galectin-3 and ECM glycoproteins might be involved in monocyte migration, given that little is known about the mechanisms by which monocytes move through a complex meshwork of proteins and carbohydrates such as the ECM. We show that monocytes migrate through distinct mechanisms that involve galectin-3 combinations with laminin and fibronectin. Interestingly, we found that macrophages migrate only in response to galectin-3-fibronectin combination.

## 2. Materials and Methods

### 2.1. Cell Preparation

Human monocytes were isolated from healthy volunteers as described elsewhere [5]. This study was approved by the National Research Ethics Committee (CONEP; document number 10461/2006), and a written informed consent was obtained from all the volunteers before their inclusion in the study. The viability of monocytes was determined by trypan blue exclusion and was >98%. Macrophages were prepared as described [6].

### 2.2. Human Recombinant Galectin-3 (hrGal-3) Preparation

 hrGal-3 and the carboxyl-terminal domain fragment of Gal-3 (truncated galectin-3 (TGal-3)) were prepared as described previously [7]. Recombinant Gal-3 was further purified by affinity chromatography on detoxi-gel beads to eliminate lipopolysaccharide (LPS) contamination, according to the manufacturer's recommendations (Pierce Chemical Co., USA). The lectin purity was analyzed by sodium dodecyl sulfate-polyacrylamide gel electrophoresis (SDS-PAGE), and protein concentration was determined using a commercial kit (BCA protein assay kit, Pierce). Biotinylation of hrGal-3 and TGal-3 with Sulfo-NHS-LC-biotin (Pierce) was performed according to the manufacturer's recommendations.

### 2.3. Flow Cytometry

Human monocytes (1 × 10^6^) were incubated or not with biotinylated-hrGal-3 (25 *µ*g/ml) for 1 h at 4°C. In some assays, biotinylated-hrGal-3 samples were previously incubated with 10 mM lactose or sucrose (Sigma) for 16 h at 4°C. Cells were washed and then incubated, for 45 min at 4°C in the dark, with FITC-conjugated streptavidin (1 : 1000; Pierce), PE-conjugated mouse anti-human CD14 monoclonal Ab (Caltag Laboratories, USA), or IgG isotype control (10 *µ*g/mL; Caltag Laboratories, USA). These antibodies were diluted in the presence of rabbit serum. Cells were acquired using a FACsort system (Becton Dickinson immunocytometry Systems, USA), and data were analyzed using the FlowJo V7.2.5 software.

### 2.4. Confocal Microscopy

 Monocytes (1 × 10^6^) were added into a 24-well culture plate (Corning Incorporated Costa, USA), containing glass slides, where they were maintained for 2 h at 5% CO_2_ and 37°C. Cells were incubated for 1 h at 4°C with biotinylated hrGal-3 (25 *µ*g/mL) or in RPMI only. In some assays, biotinylated hrGal-3 was previously incubated with 10 mM lactose or sucrose for 16 h at 4°C. Cells were washed twice with PBS and incubated, for 40 min at 4°C in the dark, with Alexa fluor 594-conjugated streptavidin (Molecular Probes Eugene, USA) and FITC-conjugated mouse anti-human CD14 monoclonal Ab (Caltag Laboratories, USA) or IgG isotype control (10 *µ*g/mL; Caltag Laboratories, USA). These antibodies were diluted in the presence of rabbit serum. After fifty washes using PBS, cells were fixed with 2% paraformaldehyde at room temperature for 20 min. Coverslips were mounted with Fluoromount-G, and images were acquired using a Leica TCS SP5 confocal microscope. 

### 2.5. Cell Migration Assays

 Monocyte migration was evaluated in a transwell system (6.5 mm-diameter polycarbonate membranes with 5.0 *µ*m-diameter pores; Corning). Cells (1 × 10^5^) were added into inserts and chemoattractants into the lower wells. Monocyte chemoattractant protein-1 (MCP-1) (100 ng/mL; PrepoTech, USA) was used as positive control and RPMI as negative. After 40 min at 37°C, nonmigrating monocytes were wiped off the insert, and the migrating cells present on the lower side of the membrane were fixed with 70% methanol and stained with HEMA3 (Biochemical Sciences, USA). The number of migrating cells was counted in five fields for each condition, assayed in triplicate. 

Assays were done using soluble or immobilized hrGal-3. For hrGal-3 immobilization, filters were previously treated with different concentrations of the lectin for 90 min at 37°C in a humidified atmosphere. After being washed with PBS, filters were dried and transferred to the transwell system. The basis for migration assays using immobilized molecules were established previously [8].

To evaluate the role of matrix glycoproteins in monocyte- or macrophage-induced migration by galectin-3, laminin, fibronectin, or vitronectin (Sigma) was immobilized on membranes as described above. 

Previous incubations of hrGal-3 samples with 10 mM lactose (or sucrose) for 16 h at 4°C were performed to test the involvement of the lectin carbohydrate recognition domain in its attractant activity.

### 2.6. Microplate Binding Assay

 A 96-well microplate was coated overnight at 4°C with 1 *µ*g/well of laminin or fibronectin diluted in 100 *µ*L of carbonate buffer. Wells were washed three times with PBS with 0.05% Tween 20 (PBS-T) and 3% gelatin (w/v) was added to the wells to block nonspecific binding. The plate was incubated for 2 h at 37°C washed and incubated for additional 2 h with biotinylated hrGal-3 or truncated Gal-3 (25 *µ*g/mL). In some assays, biotinylated-hrGal-3 was previously incubated with 10 mM lactose or sucrose for 16 h at 4°C. The plate was washed three times with PBS-T and incubated with streptavidin-peroxidase (1 : 500) for 1 h at 37°C. After washing three times with PBS-T, 100 *μ*L of 3,3′,5,5′-tetramethylbenzidine (TMB) substrate solution (BD Biosciences, USA) was added to each well for development of peroxidase reaction, and the optical density was measured at 450 nm.

### 2.7. Data Analysis

 Data are summarized as the mean ± SEM Results were statistically analysed using a paired *t*-test.

## 3. Results

### 3.1. Galectin-3 Binds to Glycosylated Molecules on Monocyte Surface

**Figure 1:**
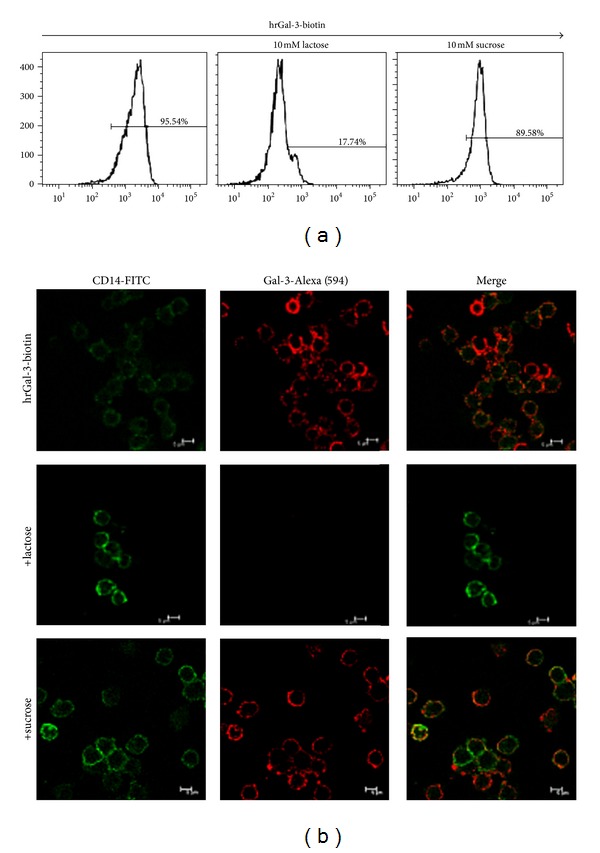
Galectin-3 binds to the surface of human monocytes, an event inhibited by the specific sugar. Human monocytes (1 × 10^6^ cells) were incubated for 1 h at 4°C with biotin-conjugated hrGal-3 (25 *µ*g/mL), in the presence of 10 mM lactose or sucrose. (a) By flow cytometry, CD14^+^ gated cells were analyzed for hrGal-3-biotin staining after reaction with streptavidin-FITC. Histograms represent the number of hrGal-3 positive cells. (b) Monocytes incubated with hrGal-3-biotin were analyzed by confocal microscopy, after incubation with anti-CD14-FITC antibody and streptavidin-Alexa Fluor 594. Results are representative from three independent assays.

To study the binding of galectin-3 to ligands on the surface of human monocytes, cells were incubated with biotinylated lectin and analyzed by flow cytometry and confocal microscopy. We also assayed the inhibition of hrGal-3 binding to the cell surface by the specific sugar, lactose. The flow cytometry results showed that 95.5% of CD14^+^ gated monocytes incubated with hrGal-3 were labeled, and in the presence of lactose, hrGal-3 binding to monocytes was significantly inhibited, as only 17.7% of cells were labeled (Figure 1(a)). On the other hand, sucrose had no significant effect on hrGal-3 binding to monocytes, as 89.6% of cells were labeled in its presence (Figure 1(a)). For confocal microscopy analysis, we labeled monocytes with anti-CD14 antibody stained with FITC, and hrGal-3 stained with Alexa594. As shown in Figure 1(b), hrGal-3 was evenly distributed on the surface of CD14-labeled monocytes. Binding of the lectin was inhibited in the presence of lactose, whereas sucrose had no effect.

These results indicate that galectin-3 binds to ligands expressed on the surface of human monocytes and that such an interaction is dependent on Gal-3 lectin binding activity.

### 3.2. Both Soluble and Immobilized Galectin-3 Induces Monocyte Migration

**Figure 2:**
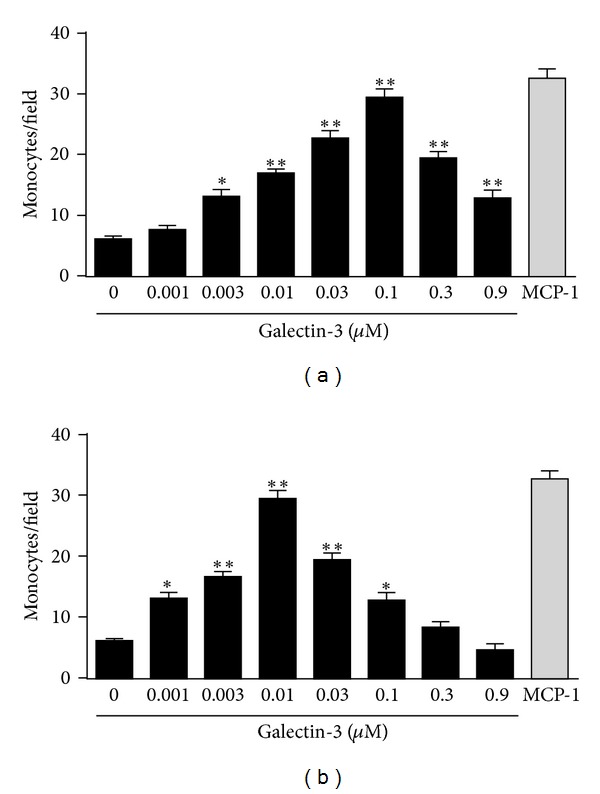
Soluble or immobilized galectin-3 induces monocyte migration in a dose-dependent manner. In transwell systems, human monocytes (5 × 10^4^ cells) were added into the inserts and were allowed to migrate in response to different concentrations (0.001–0.9 *µ*M) of soluble (a) or immobilized (b) hrGal-3 for 40 min at 37°C. The number of migrating monocytes was counted in five fields for each condition assayed in triplicate. MCP-1 (100 ng/mL) was used as positive control and medium as negative. Data are reported as mean of monocytes per field ± SEM sGal-3, soluble Gal-3; iGal-3, immobilized Gal-3. *P < 0.05 and **P < 0.01 compared to negative control. Results are representative from three independent assays.

We used transwell systems to test the attractant activity of soluble hrGal-3 (sGal-3) toward human monocytes. Soluble hrGal-3 (0.003–0.9 *µ*M) induced monocyte migration in a dose-dependent manner, yielding a typical bell-shaped dose-response curve. Maximum attractant activity was observed at 0.1 *μ*M, similar to that induced by MCP-1, a potent attractant for monocytes used as positive control (Figure 2(a)). 

To evaluate the ability of galectin-3 to induce monocyte haptotaxis, polycarbonate filters containing different concentrations of immobilized hrGal-3 (iGal-3; 0.001–0.9 *µ*M) were used in transwell systems. Immobilized hrGal-3, as soluble hrGal-3, induced monocyte migration in a dose-dependent manner. However, when compared to chemotaxis, a ten-times lower concentration of hrGal-3 (0.01 *μ*M) was sufficient to induce the maximum response, similar to that determined by MCP-1 (Figure 2(b)). These results indicate that a substrate of galectin-3, formed through its immobilization on polycarbonate filters, improves the migratory response of monocytes induced by the lectin.

### 3.3. Extracellular Matrix Glycoproteins Potentiate the Monocyte Migration Induced by Galectin-3

**Figure 3:**
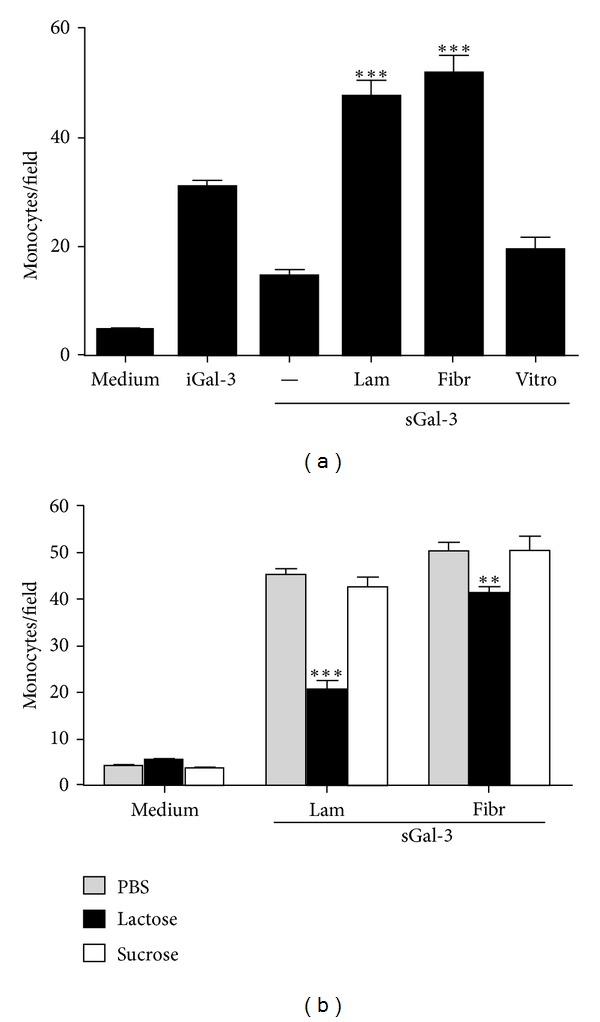
Monocyte migration induced by galectin-3 is potentiated in the presence of laminin and fibronectin, but not vitronectin. Polycarbonate filters of a transwell system were coated with laminin, fibronectin, or vitronectin as described in Section 2 and used to assay the monocyte migration induced by soluble hrGal-3 (0.01 *µ*M). Monocytes (5 × 10^4^ cells) were added into inserts and allowed to migrate for 40 min at 37°C. Medium was used as negative control and immobilized hrGal-3 as positive. The chemoattractant activity of sGal-3 in concert with laminin or fibronectin was tested in the absence (a) or presence of 10 mM lactose or sucrose (b). The number of migrating monocytes was counted in five fields for each condition assayed in triplicate. Data are reported as mean of monocytes per field ± SEM sGal-3, soluble Gal-3; iGal-3, immobilized Gal-3; Lam, laminin; Fibr, fibronectin; Vitro, vitronectin. ***P < 0.001 compared to iGal3 in (a), and ***P < 0.001 or **P < 0.01 compared to sGal-3 plus laminin or fibronectin in the presence of PBS in (b). Results are representative from three independent assays.

To verify the involvement of the extracellular matrix glycoproteins laminin, fibronectin, or vitronectin in the attractant activity of galectin-3, we evaluated the monocyte migration in response to hrGal-3 stimulus in the presence of gradients of these molecules. To this end, 0.01 *μ*M hrGal-3 was used given that this concentration determined the maximum response of monocytes in our previous assay, as described above. As shown in Figure 3(a), hrGal-3 induced monocyte migration both when immobilized or not on filters. However, the response was significantly higher upon immobilization, confirming the previous results. When soluble hrGal-3 was assayed in the presence of immobilized laminin or fibronectin, but not vitronectin, we observed a monocyte migration significantly higher than that obtained in response to soluble hrGal-3 alone (Figure 3(a)). Soluble or immobilized laminin, fibronectin, and vitronectin, in the absence of galectin-3, were not able to induce monocyte migration (data not shown).

To investigate if the carbohydrate recognition domain (CRD) of galectin-3 is involved in the monocyte migration in response to the lectin in combination with laminin or fibronectin, the assays were performed using soluble hrGal-3 previously incubated with sugars. In agreement with the results described above (Figure 3(a)), soluble hrGal-3 induced monocyte migration in the presence of immobilized laminin or fibronectin, showing similar responses in both conditions. Lactose significantly inhibited monocyte migration induced by soluble hrGal-3 alone (not shown) or in combination with laminin or fibronectin. A nonspecific sugar, sucrose, did not affect the cell migration induced by hrGal-3 in combination with both glycoproteins (Figure 3(b)).

These results indicate that monocyte migration induced by galectin-3 is potentiated in the presence of laminin and fibronectin, but not vitronectin. The CRD of the lectin is implicated in the monocyte migration induced by galectin-3 in combination with laminin or fibronectin.

### 3.4. Galectin-3 Binds to Laminin but Not to Fibronectin, through Its Carbohydrate Recognition Domain

**Figure 4:**
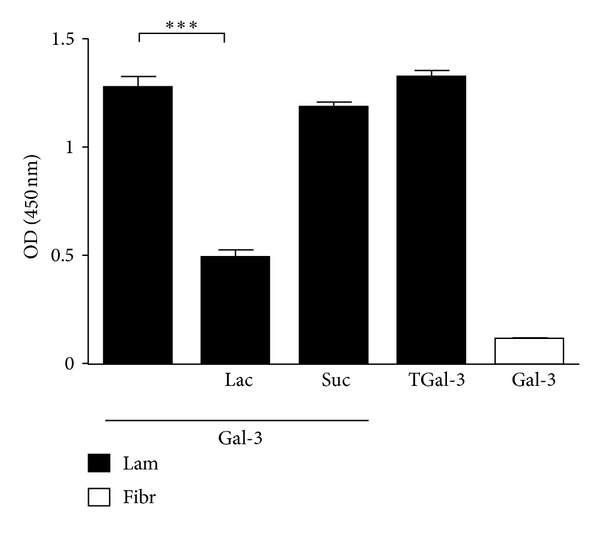
Galectin-3 binds to laminin through its carbohydrate recognition domain. A 96-well microplate was coated with 1*µ*g/well of laminin or fibronectin and incubated for 2 h with biotinylated hrGal-3 or truncated Gal-3 (25 *µ*g/mL). In some assays, biotinylated hrGal-3 was previously incubated with 10 mM lactose or sucrose. The signal was generated by streptavidin-peroxidase using 3,3′,5,5′-tetramethylbenzidine as substrate. Gal-3, galectin-3; TGal-3, truncated galectin-3; Lam, laminin; Fibr, fibronectin, Lac, lactose; Suc, sucrose. ***P < 0.001 compared to Gal-3 in the absence of sugar. Results are representative from two independent assays.

To investigate if galectin-3 binds to laminin and fibronectin, we performed galectin-3 binding assays to laminin- or fibronectin-coated microplates. Both hrGal-3 and its truncated form, which lacks the N-terminal domain, bound to laminin. On the other hand, hrGal-3 did not bind to fibronectin. Confirming our previous results, lactose inhibited hrGal-3 binding to laminin, whereas sucrose had no effect on this binding (Figure 4).

These results indicate that galectin-3 binds to laminin but not fibronectin, and that such an interaction is dependent on the carbohydrate recognition domain of the lectin.

### 3.5. Fibronectin, but Not Laminin, Contributes to Macrophage Migration Induced by Galectin-3

**Figure 5:**
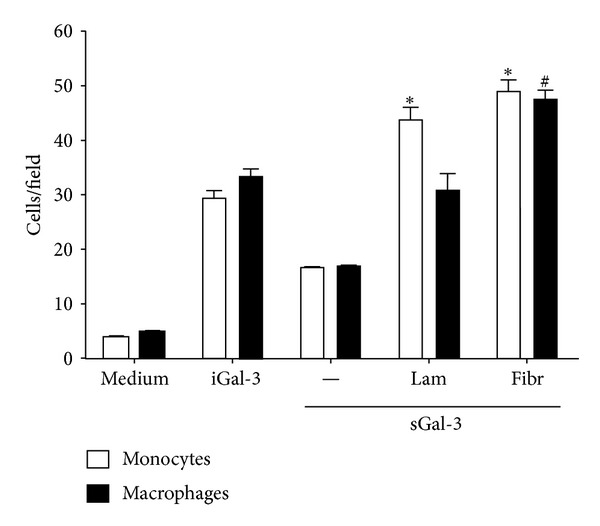
Interactions established between Gal-3 and laminin or fibronectin contribute to migration of monocytes and macrophages. Polycarbonate filters of a Transwell System were coated with laminin or fibronectin as described in Section 2 and used to assay cell migration induced by soluble hrGal-3 (0.01 *µ*M). Monocytes or macrophages (5 × 10^4^ cells) were added into inserts and allowed to migrate for 40 min at 37°C. Medium was used as negative control and soluble or immobilized hrGal-3 as positive. The number of migrating cells was counted in five fields for each condition assayed in triplicate. Data are reported as mean of cells per field ± S.E.M. sGal3, soluble Gal3; iGal3, immobilized Gal3; Lam, laminin; Fibr, fibronectin. *P < 0.05 compared to iGal3 assayed with monocytes and ^#^ *P* < 0.05 compared to iGal3 assayed with macrophages. Results are representative from two independent assays.

Upon leaving the vasculature, monocytes undergo further maturation and differentiate into macrophages. To evaluate if the mechanisms by which galectin-3 induces the migration of monocytes are also involved in the migratory response of macrophages to this lectin, the migration assays were performed using human monocyte-derived macrophages (Figure 5). As observed for monocytes, both soluble and immobilized hrGal-3 induced the migration of macrophages, and the response was higher toward immobilized lectin. When compared to the macrophage migration toward immobilized hrGal-3 alone, the presence of fibronectin potentiated the response induced by the lectin, since it was 60% higher, whereas the association of hrGal-3 with laminin did not. This behavior differed from that observed for monocytes, as their migration was improved when soluble hrGal-3 was combined with both glycoproteins, laminin, or fibronectin.

These results show that macrophage migration induced by galectin-3 is potentiated by fibronectin but not by laminin.

## 4. Discussion

Recruitment of monocytes to inflammatory sites involves a series of sequential attachments and detachments to extracellular matrix proteins in response to an attractant gradient [1]. We attempted to gain further insights into the mechanisms involved in monocyte migration and found that interactions between galectin-3 and components of the extracellular matrix are differentially involved in the process. Thus, while monocytes migrate in response to associations/combinations of galectin-3 and laminin or fibronectin, monocyte-derived macrophages have a distinct behavior and migrate only in response to galectin-3 combined with fibronectin.

Monocyte movement, as reported for other leukocytes, is driven by a gradient of an attractant molecule [1]. Previous studies have shown that both monocytes and macrophages migrate toward galectin-3 [5], but the molecular interactions between these leukocytes and the vessel wall or the perivascular tissue, essential to the migratory process, were not investigated. We therefore expanded the characterization of the monocyte migratory process in response to galectin-3.

First, we studied the binding of galectin-3 to the surface of monocytes, and we showed that binding of galectin-3 to monocytes is dependent on the carbohydrate recognition domain of the lectin, in agreement with previous work that demonstrated the involvement of the CRD in monocyte migration [5]. Furthermore, we verified that galectin-3 ligands are evenly distributed on the monocyte surface. 

By virtue of its potential multivalence, galectin-3 can establish concomitant interactions with glycans on different substrates, such as the extracellular matrix and monocyte surface. Therefore, it is conceivable that monocyte migration induced by galectin-3 occurs through haptotaxis, a mechanism in which cell migration is driven by an immobilized gradient of the attractant molecule, which binds to substrates such as the ECM components on the surface of endothelial cells, in the basement membrane, and in the connective tissue [9, 10]. ECM components include laminin, fibronectin, vitronectin, collagen, and sulfated proteoglycans [11]. The glycoprotein fibronectin is present in the interstitial space and plays a major role in cell adherence to the tissue [12–14]. This molecule is also found in the basement membrane along with laminin, which is the most abundant glycoprotein in this layer [15, 16].

In our study, using transwell systems, we showed that monocytes migrated in response to both soluble and immobilized galectin-3. However, when immobilized, lower concentrations of the lectin determined higher responses. We then analyzed the involvement of extracellular matrix glycoproteins in monocyte migration in response to galectin-3 and observed significantly higher migratory responses in the presence of laminin and fibronectin when compared to those determined by both soluble and immobilized galectin-3 alone. Vitronectin, on the other hand, did not affect the monocyte migration toward galectin-3. Migration induced by galectin-3 in combination with laminin or fibronectin was dependent on the lectin CRD. These findings suggest that galectin-3 interacts selectively with specific molecules present in the extracellular matrix. 

As reported previously, the interactions of galectin-3 with extracellular matrix components are involved in cell adhesion. The lectin promotes adhesion of neutrophils to laminin and fibronectin [17] and enhances the adhesion of a tumor cell line to these molecules as well as to vitronectin [18]. Although the findings obtained by these groups demonstrate the adhesive role of galectin-3, they cannot be directly extrapolated to cell migration, as after adhesion to the vascular endothelium, cells need to breach this vascular barrier to reach the underlying tissue, and this is achieved after an appropriate activation of intracellular signaling [1]. 

We showed that although both laminin and fibronectin potentiated the monocyte migration induced by galectin-3, the lectin was able to bind to laminin but not to fibronectin. Galectin-3 laminin associations are dependent on the lectin's carbohydrate-binding activity, as they were selectively inhibited by lactose. Furthermore, the NH2-terminally truncated form of galectin-3 generated by collagenase digestion of the lectin, which retains carbohydrate binding ability, also bound to laminin. Therefore, it is plausible that *in vivo* galectin-3, by binding to carbohydrate residues of laminin, can form a substrate to drive the transmigration of monocytes across the endothelium and their movement through the extravascular tissue. By cross-linking appropriately glycosylated receptors on opposing surfaces, namely, the monocyte surface and laminin molecules on the ECM, the lectin could bridge monocytes to laminin. On the other hand, a mechanism independent of physical associations between galectin-3 and fibronectin may be involved in monocyte movement along a fibronectin-rich substrate. As previously reported for neutrophils [17], monocytes may bind to fibronectin as a consequence of a cell activation process induced by galectin-3. Indeed the lectin, upon binding to extracellular domains of transmembrane proteins, can either upregulate their expression or modulate the binding affinity and clustering of these proteins to their extracellular ligands [17, 19]. We did not detect any difference in the surface levels of the integrins CD11b and CD11c and of L-selectin in galectin-3-stimulated monocytes. Likewise, the levels of *ICAM*, *VCAM*, *VLA-4*, *LFA-1*, *PSGL-1,* and *Mac-1* mRNA transcripts did not increase upon monocyte stimulation with galectin-3 (data not shown). Therefore, monocyte migration on a fibronectin substrate is most likely due to the redistribution of adhesion molecules on the cell surface and/or to increased ligand binding affinity or avidity to fibronectin induced by galectin-3. Evidently, the same mechanisms may also contribute to migration on a laminin substrate. Considering that both laminin and fibronectin are recognized by a number of cell surface receptors of the integrin family [3, 20–22] and that integrin molecules are included among surface receptors for galectin-3 on different cell types, including monocytes/macrophages [23, 24], these adhesion molecules are potentially involved in monocyte movement induced by galectin-3 along laminin and fibronectin substrates. Indeed, galectin-3-induced modulation of integrin clustering/redistribution has been previously demonstrated to regulate cell adhesion or motility on the ECM [25–27]. Interestingly, a functional synergistic loop between *β*1-integrins and galectin-3 has been recently demonstrated. While galectin-3 expression was induced by the introduction of the integrin *β*1 in GE11 epithelial cells, galectin-3 expression, in turn, enhanced *β*1 integrin-mediated cell adhesion to fibronectin and laminin, as well as cell migration [28].

On their recruitment to sites of injury, monocytes leave the blood stream and after crossing the vessel wall, reach the extravascular space, and eventually mature into macrophages. In the connective tissue, macrophages contact the extracellular matrix, which modulates their maturation [29]. Present in the ECM, fibronectin contributes to the monocyte/macrophage differentiation process and has been described as an important function modulator of macrophages [30]. In the present study, we demonstrated that the migratory response of monocyte-derived macrophages toward galectin-3 differs from monocytes, as macrophage migration is potentiated when the lectin is combined with fibronectin but not laminin. 

In general, haematopoietic cell differentiation is determined by finely regulated signals acting on gene expression and leading to the achievement of terminally differentiated phenotypes within the proper cell lineages [31]. Cellular modifications occur as a consequence of specific leukocyte maturation, and several proteins are differentially expressed on the cell surface [31]. Noteworthy is the fact that when monocytes differentiate into macrophages, galectin-3 expressed on the cell surface is upregulated [32]. In addition, monocyte-derived macrophages differentiated into different phenotypes have been shown to exhibit specific patterns of galectin-3 expression [33]. Based on the distinct migratory response presented by monocytes and macrophages in our study, we cogitate that the modulation of cell surface molecules might affect how migrating cells perceive and discriminate their extracellular matrix ligands. It is conceivable that galectin-3 highly expressed on the macrophage surface would indirectly mediate their contact with fibronectin, contributing to their interstitial migration.

In summary, our findings provide further elucidation on the underlying mechanisms involved in monocyte migration. These data show that monocytes migrate through selective interactions with the extracellular matrix driven by galectin-3. Both ECM glycoproteins, laminin and fibronectin, would be involved in monocyte migration, whereas only fibronectin would be implicated in macrophage migration. We speculate that during inflammatory response, mononuclear phagocytes may migrate towards injured sites through discriminating molecular interactions mediated by different attractants, including now galectin-3. A better understanding of the mechanisms underlying galectin-3-mediated cell migration and cell matrix interactions is critical to examine the role of the lectin as a potential therapeutic target in inflammatory disorders.

## References

[1] Imhof BA, Aurrand-Lions M (2004). Adhesion mechanisms regulating the migration of monocytes. *Nature Reviews Immunology*.

[2] Ganiko L, Martins AR, Freymüller E, Mortara RA, Roque-Barreira MC (2005). Lectin KM^+^-induced neutrophil haptotaxis involves binding to laminin. *Biochimica et Biophysica Acta*.

[3] de Toledo KA, Bernardes ES, Baruffi MD, Roque-Barreira MC (2007). Neutrophil haptotaxis induced by mouse MNCF: interactions with extracellular matrix glycoproteins probably contribute to overcoming the anti-inflammatory action of dexamethasone. *Inflammation Research*.

[4] Rabinovich GA, Rubinstein N, Toscano MA (2002). Role of galectins in inflammatory and immunomodulatory processes. *Biochimica et Biophysica Acta*.

[5] Sano H, Hsu DK, Yu L (2000). Human galectin-3 is a novel chemoattractant for monocytes and macrophages. *Journal of Immunology*.

[6] Franco LH, Wowk PF, Silva CL (2008). A DNA vaccine against tuberculosis based on the 65 kDa heat-shock protein differentially activates human macrophages and dendritic cells. *Genetic Vaccines and Therapy*.

[7] Hsu DK, Zuberi RI, Liu FT (1992). Biochemical and biophysical characterization of human recombinant IgE- binding protein, an S-type animal lectin. *Journal of Biological Chemistry*.

[8] Webster GF, Leyden JJ (1980). Characterization of serum-independent polymorphonuclear leukocyte chemotactic factors produced by Propionibacterium acnes. *Inflammation*.

[9] Rot A (1992). Endothelial cell binding of NAP-1/IL-8: role in neutrophil emigration. *Immunology Today*.

[10] Qi M, Ikematsu S, Maeda N (2001). Haptotactic migration induced by midkine: involvement of protein-tyrosine phosphatase *ζ*, mitogen-activated protein kinase, and phosphatidylinositol 3-kinase. *Journal of Biological Chemistry*.

[11] Hynes RO (2009). The extracellular matrix: not just pretty fibrils. *Science*.

[12] Dastych J, Costa JJ, Thompson HL, Metcalfe DD (1991). Mast cell adhesion to fibronectin. *Immunology*.

[13] Midwood KS, Mao Y, Hsia HC, Valenick LV, Schwarzbauer JE (2006). Modulation of cell-fibronectin matrix interactions during tissue repair. *Journal of Investigative Dermatology Symposium Proceedings*.

[14] Anceriz N, Vandal K, Tessier PA (2007). S100A9 mediates neutrophil adhesion to fibronectin through activation of *β*2 integrins. *Biochemical and Biophysical Research Communications*.

[15] Arroyo AG, Iruela-Arispe ML (2010). Extracellular matrix, inflammation, and the angiogenic response. *Cardiovascular Research*.

[16] Järveläinen H, Sainio A, Koulu M, Wight TN, Penttinen R (2009). Extracellular matrix molecules: potential targets in pharmacotherapy. *Pharmacological Reviews*.

[17] Kuwabara I, Liu FT (1996). Galectin-3 promotes adhesion of human neutrophils to laminin. *Journal of Immunology*.

[18] Matarrese P, Fusco O, Tinari N (2000). Galectin-3 overexpression protects from apoptosis by improving cell adhesion properties. *International Journal of Cancer*.

[19] Demetriou M, Granovsky M, Quaggin S, Dennis JW (2001). Negative regulation of T-cell activation and autoimmunity by Mgat5 N-glycosylation. *Nature*.

[20] Johansson S, Svineng G, Wennerberg K, Armulik A, Lohikangas L (1997). Fibronectin-integrin interactions. *Frontiers in Bioscience*.

[21] Stipp CS (2010). Laminin-binding integrins and their tetraspanin partners as potential antimetastatic targets. *Expert Reviews in Molecular Medicine*.

[22] Santos-de-Oliveira R, Dias-Baruffi M, Thomaz SMO, Beltramini LM, Roque- Barreira MC (1994). A neutrophil migration-inducing lectin from *Artocarpus integrifolia*. *Journal of Immunology*.

[23] Dong S, Hughes RC (1997). Macrophage surface glycoproteins binding to galectin-3 (Mac-2-antigen). *Glycoconjugate Journal*.

[24] Dumic J, Dabelic S, Flögel M (2006). Galectin-3: an open-ended story. *Biochimica et Biophysica Acta*.

[25] Lagana A, Goetz JG, Cheung P, Raz A, Dennis JW, Nabi IR (2006). Galectin binding to Mgat5-modified N-glycans regulates fibronectin matrix remodeling in tumor cells. *Molecular and Cellular Biology*.

[26] Friedrichs J, Manninen A, Muller DJ, Helenius J (2008). Galectin-3 regulates integrin *α*2*β*1- mediated adhesion to collagen-I and -IV. *Journal of Biological Chemistry*.

[27] Kariya Y, Kawamura C, Tabei T, Gu J (2010). Bisecting GlcNAc residues on laminin-332 down-regulate galectin-3-dependent keratinocyte motility. *Journal of Biological Chemistry*.

[28] Margadant C, van den Bout I, van Boxtel AL, Thijssen VL, Sonnenberg A (2012). Epigenetic regulation of galectin-3 expression by *β*1 integrins promotes cell adhesion and migration. *The Journal of Biological Chemistry*.

[29] Jacob SS, Shastry P, Sudhakaran PR (2002). Monocyte-macrophage differentiation in vitro: modulation by extracellular matrix protein substratum. *Molecular and Cellular Biochemistry*.

[30] Sudhakaran PR, Radhika A, Jacob SS (2007). Monocyte macrophage differentiation in vitro: fibronectin-dependent upregulation of certain macrophage-specific activities. *Glycoconjugate Journal*.

[31] Minafra L, Di Cara G, Albanese NN, Cancemi P (2011). Proteomic differentiation pattern in the U937 cell line. *Leukemia Research*.

[32] Liu FT, Hsu DK, Zuberi RI, Kuwabara I, Chi EY, Henderson WR (1995). Expression and function of galectin-3, a *β*-galactoside-binding lectin, in human monocytes and macrophages. *American Journal of Pathology*.

[33] Novak R, Dabelic S, Dumic J (2012). Galectin-1 and galectin-3 expression profiles in classically and alternatively activated human macrophages. *Biochimica et Biophysica Acta*.

